# The History of the Introduction of the Concept of Depression Into China

**DOI:** 10.3389/fpsyt.2022.889329

**Published:** 2022-06-02

**Authors:** Jike Bai, Jun Li, Daqing Zhang

**Affiliations:** ^1^Global Health and Social Medicine, Harvard Medical School, Boston, MA, United States; ^2^School of Medical Humanities, Peking University, Beijing, China

**Keywords:** depression, early stage, input, introduction, public cognition

## Abstract

This is a thematic historical study on the historical construction of the concept of depression in early modern China. Using an external historical research method, through the analysis of newspaper stories, drug advertisements, and medical texts (textbooks and reference books), it presents the sociocultural context of depression in the late Qing Dynasty and early Republican period and depicts the germination and evolution of the depression from a hazy and ambiguous concept in the late Qing Dynasty to a clear and complete disease entity of Western medicine, at least in the Chinese pharmaceutical market in the 1920s. This article examines the three internal logical clues in the localization of depression in China, namely, (1) the transformation of the disease from a symptom (the symptom of a disease) to a disease (an independent disease entity); (2) the pathological mechanism of depression was first made from the perspective of Traditional Chinese Medicine—“caused by stagnation of liver qi,” which was joined later by the pathological mechanism of Western medicine—“caused by brain dysfunction”; (3) the introduction of the knowledge of “depression” presents a pattern of “cross-fertilization” between the West and the East. This study also examines the cultural imagery of depression during its early introduction to China and finds the three stereotypes of the manifestation of depression among the then Chinese public, namely, a feminized disease, a disease that afflicted the intellectual youth who were worried about the country, and the association between the disease and the morbid and distorted state of life of the upper-class literary youth.

## The Emergence of the Concept of Depression

The concept of depression spread to China in the late Qing Dynasty. Actually, it was the concept of melancholia, not depression, that was introduced into China, and it was often translated as *Youyu Zheng (in Pinyin) or 忧郁症 (in Chinese characters)*. Its debut was in the *North China Herald & Supreme Court and Consular Gazette* on 26 May 1893, entitled *Victim of Panama* (1), a short story about a man in London, Charles, sent to hospital due to severe depression.

**FIGURE 1:**
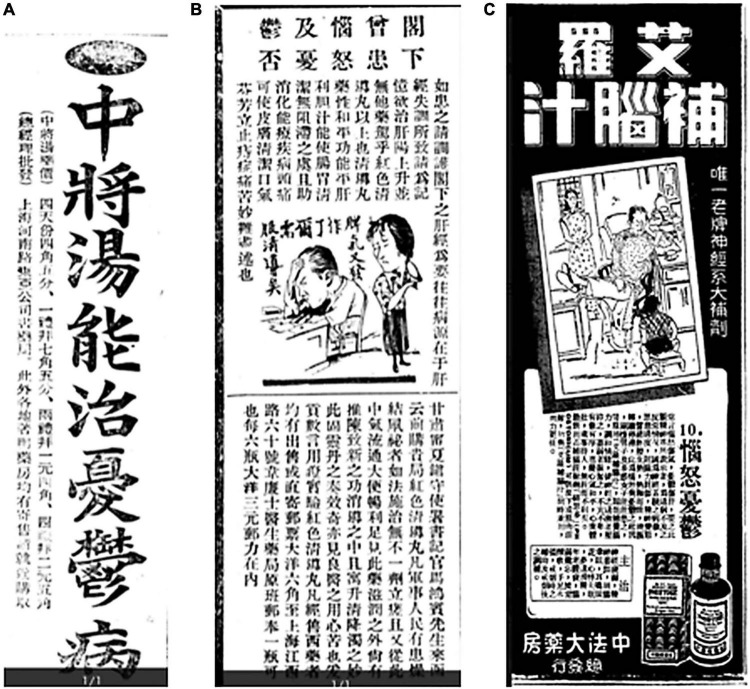
**(A)** Advertisement of *Zhongjiang Tang*. Source: *Times* (时报), 1 April 1913. **(B)** Advertisement of the Red Qingdao pill. Source: *Republic of China Daily* (国民报), 31 March 1925. **(C)** Advertisement of Ailuo Brain Tonic. Source: *The News* (新闻报), 29 November 1940.

As early as the beginning of the Republic of China, advertisements for antidepressants appeared in newspapers. The advertisement for *Zhongjiang Tang (中将汤)*, a drug for melancholia sold by the East Asia Company Pharmacy located on Henan Road in Shanghai, first appeared on page 11 of *Ta Kung Pao (大公报)*, a newspaper published in the northern city of Tianjin, in 1911 (2), and later in the healthcare columns of the *Shibao 时报 (or Times)* on 1 April 1913 (page 11, Figure 1A) (3); 4 April 1913 (page 14) (4); and 13 August 1913 (page 4); and in page 16 of *Shen Bao 申报 (or Shanghai News)* (5) on 15 April 1914 (6). Analysis of the content and the frequency of the advertisements proves that this drug had been manufactured and sold by a pharmaceutical company in China at that time and that there was a market for antidepressants. We could infer that the concept of depression had appeared in China then.

In the 1920s, Western medicines for depression began to appear in the Chinese market. The explanation of the pathological mechanism of depression was one of traditional Chinese Medicine (TCM)—“stagnation of *Qi* due to the depression of the liver” in the beginning, and was replaced by “neuropsychiatric disorder and brain function depletion.” In other words, there was a transition of explanation of the mechanism from an inward perspective of TCM to an integrated perspective of TCM and Western medicine. The advertisements for the Red Qingdao Pill, a drug made by Dr. Williams’ Pharmacy for anger and melancholy, were published in the *Industrial and Commercial News (Gong Shang Xinwen, 工商新闻)* on 10 (7), 17 (8), and 31 January (page 12) (9), *the Shibao 时报 (or Times)* on 29 March (page edition) (10) and 31 March (page 8) (11), *the News (Xinwen Bao, 新闻报)* on 29 March (page 6) (12), and 24 December (page 16) (13), and *the National News, Guomin Bao 国民报* (14, 15) on 31 March in 1925 (Figure 1B). The advertisement reads, “Have you ever suffered from anger and melancholia? If you have, please regulate your liver meridian, as they are often caused by the imbalance of the liver meridian.” The beginning sentence of the advertisement implies that it is a drug for the disease of bad emotions, which equals melancholia, together with anger, as a disease. In traditional Chinese culture, emotion was rarely considered a disease. At most, it was considered as a sign. Therefore, when the mechanism of this Western medicine was explained, the TCM rhetoric “liver meridian disorder, and rising of liver Yang” was cited. Such an approach of grafting Western drugs on TCM reflects the penetration of depression, a disease in Western medicine in TCM in the early days of the Republic of China. It was probably a cleverly devised marketing means to make the medicine more acceptable as well.

In the late period of the Republic of China, the explanation of the pathology of melancholia gradually showed the integration of TCM and modern medicine. The TCM pathological perspective of “the liver meridian damaged by melancholia” and the modern medical knowledge, such as nervous system disorder, the physiological function of brain, psychopathy, were put together to explain the pathology of depression. An advertisement of the Sino-French Pharmacy published on the third page of *the News* on 29 November 1940, which was entitled the *Ailuo Brain Tonic: The Only Time-tested Tonic for the Neurological System*, wrote:

It is said that anger hurts Qi and melancholia the liver. The cause of anger and melancholia is excess thinking affecting the nervous system, resulting in mental exhaustion and psychopathy. It is like mental frictions that generate heat, extremely hot and dry, that stagnates the functioning of the mind. Self-willed men and sentimental women often suffer from these two diseases. If they could take the drug, they could enhance their brain power and harmonize their emotions. Those who are overexcited can suppress over-excitement, and those who are weak in their mind can strengthen it. They could become neither arrogant, nor humble in their demeanor, well-organized, tranquil, and tolerant. They could restore their physical health, and socialize with others properly (16) (Figure 1C).

It could be seen that the Ailuo Brain Tonic mentioned in the advertisement was a drug for anger and depression. The advertisement also pointed out that depression was a disease of the neuropsychiatric system caused by overthinking, and the people susceptible to it were self-willing, paranoid men and sentimental women.

## Early Input of the Concept of Depression from Abroad

Most early introductions to depression in China appeared in English-language newspapers, with *The Shanghai Times*, *The China Press*, and *The North-China Herald* as the top three. There were three categories of depression-related information in these newspapers, namely, stories on depression care or suicide due to depression; information about relevant films and books; and information about professional knowledge. Among them, the latter two categories dominate, mainly published in the culture and life columns of the newspapers. Examples of each category are described below.

### News on Depression Care or Suicide Due to Depression

On 26 September 1911, *The China Press* reported the case of a young German living in Kobe, who had just turned 26, cut his throat and wrist due to depression and eventually died from excessive blood loss (17). On 13 May 1914, *The China Press* reported the news that Mr. Warner, consul of the United States Consulate in Harbin, committed suicide with a pistol in the hospital and pointed out that he was suffering from depression, a mental illness, for a long time (18). The 1937 report of *The North-China Herald* explains to readers the serious consequences of depression through the case of suicide of a female patient suffering from mild depression and nervousness, and conveys to readers that depression is a hereditary but often progressive disease through the words of the patient’s doctor (19). The news of suicide or hospitalization due to depression at least taught the Chinese readers that depression was a mental illness and could have serious consequences like suicide.

### Information on Films and Books

*The North-China Herald*’s culture and life column reported in 1929 on a new, academic, and artistic interpretation of Dürer’s famous painting *Melencolia I* (20), and in 1933 on Dr. Norman’s academic discovery that people with Melancholia would lose their sense of pain, with the several cases Norman received as the evidence (21). In February 1914, *The China Press* introduced and highly praised Burton’s *The Anatomy of Melancholy*, which was also the first introduction to the book in China (22). On 4 August 1937, the black-humor comedy “Oh Doctor!” based on the 1923 novel of the same name by Harry Leon Wilson, a depression-themed thriller, was shown at the Shanghai Theater. The film tells the story of a patient with depression who lived in remorse for bargaining before recovering his inheritance and losing his inherent rights due to excessive intake of pills. *The China Press* reported the showing of the film on the same day (23). A short story of the newspaper on 11 October 1938, reported that Pokers were invented in 1390, with its original purpose to amuse King Charles VI of France, who suffered from melancholia (24). On 15 November 1938, the newspaper reported that the Kawasaki Song and Dance House in Tokyo, Japan, opened in 1932, would close at the end of the year due to the impact of the Sino-Japanese War and the depression of business and that the girls working in the house, who used to live a life of singing and dancing, had to face a bleak future—to work in factories or marry and thus got depressed and suffered from melancholia (25).

### Professional Knowledge

Medical information and research advances related to depression, mostly written by doctors. *The Shanghai Times*’ published in its 15 January 1930, column, *Your Health and Your Brain*, a story entitled *Melancholia*, written by Dr. Leland B. Alford (26). The author emphasizes the importance of mental health for assessing a person’s overall health in clinical practice. The author also points out that individuals with melancholia often complain of physical discomfort. However, the physician could not detect any physical illness that could cause such complaints despite the assertion of the patient for the existence of serious diseases underlying such discomforts. On the contrary, patients with serious physical disorders rarely have similar complaints. The author continues to emphasize, “To be sure, the symptoms of melancholia are due to mental disorders. The patient’s mind becomes so sensitive that it converts many normal physical feelings into unpleasant ones. The fear of disease is further amplified by excessive attention to their physical health.” The author also points out that this painful feeling, although real and profound, may be imagined and caused by the disruption of the balance of mental activity. An accurate description of the process of depression can be obscure and vague, but the author believes that the pain it causes can be simply imagined. Therefore, it may be extremely difficult to distinguish between depression and physical disease by simply relying on the typical symptoms caused by “imbalance.” Alford uses “hypochondria” to refer to depression in this article, but with the author’s mentioning of the symptoms of fear, hypochondria, imagination in the article, the disease he discusses is likely to encompass today’s hypochondria disorder. *The Shanghai Times* clarified the relationship between melancholia and depression in an article written by Dr. Lago Galdston published in the *Daily Health Talk* column on 22 November 1934. He believes that the ancient name melancholia suggests that depression was thought to be caused by black bile and was considered an excessive state. Furthermore, the black bile explanation implies that he believes the mental state of depression is caused by aberrations of chemicals and anatomy, or dysfunction of the body, rather than subtle, uncertain psychological causes. In other words, depression was thought to be a complex mental disorder caused by physiological disorders in ancient times. He also believes that depression is not a special mental disorder, but a mental symptom of many mental diseases that occur in both sexes, although likely to be more common in women and that people are prone to suffer it in their 40s and 50s and women would have an earlier onset than men. He states that patients with depression would complain of neurological symptoms, show a loss of interest, a significant loss of memory and attention, cry frequently, have unwarranted worry and fear, and some patients would develop delusions (27). It can be seen that his understanding of depression is very similar to that of contemporary people, both in terms of demographic factors and in the interpretation of clinical manifestations. In the next day’s newspaper, Dr. Galdston continued to introduce the latest research on climacteric melancholia, which used a new treatment of estrogen in a controlled experiment to overturn the previous theory that climacteric depression was caused by typical personality characteristics, such as seriousness, rigidity, lack of humor, and over-cautiousness and lifestyle factors (28). In the news of 19 August 1939, *The Shanghai Times* reported that Dr. F. T. Thorpe of West Knight Hospital had discovered a new type of depression called senile depression and published his discovery in the *British Medical Journal* (29).

There were few psychiatry books available in modern China. Most of them were translated from Japanese or European works, and the works of local psychiatrists were also influenced by Japanese, European, and American scholars.

The works of Japanese psychiatrists were introduced into China. Examples include *The Essentials of Psychiatry 精神病学集要* written by Ying Shuzo 吴秀三 published in 1916 (30), *Diagnosis and Treatment of Psychiatric Diseases* 精神病诊断及治疗学 by Miyake Koichi *三宅矿一* and Takaburo Matsumoto *松本高三郎* in 1921 (31), *Outlines of Psychiatry 精神病学精要* by Miyake Koichi in 1934 (32) (and reprinted in 1940) (33), and *Summary of Psychiatry 精神病学馀沥* by the same author in 1935 (34), Minor Psychiatry 小精神病学 by Sugita Naoyuki/Naoki Sugda 杉田直树 in 1933 (35), and *Lecture Notes on Psychiatry 精神病学讲义* by Kijuro Uematsu in 1942 (36). Miyake Koichi mentions in his works that depression is associated with narcolepsy encephalitis, suggesting that encephalitis may lead to depression. Moreover, he believes that depression may be inheritable in families, and there is a linear regression relationship between typical depression and early-onset dementia. The process of diagnosis and treatment of patients with depression described by him in *Tongren Medicine* in 1931 includes cerebrospinal fluid as an examination item (37).

The authors of the article also found that cerebrospinal fluid examination was a routine examination item in the medical records of patients with depression treated in modern China in Nanjing Brain Hospital (for details, see another article by the authors). It can be seen that the theories of Japanese scholars have had an impact on the diagnosis and treatment of depression in modern China. The contents related to depression, such as bipolar disorder, and depressive state in the *Psychiatric Lecture Notes of Peiping Medical College* were deeply influenced by Japanese psychiatric works.

China’s first monograph on psychiatry *A Brief Introduction to Mental Illness* (38), co-translated by P. B. Cousland and M. J. Chu from the *Insanity in Everyday Practice* by E. G. Younger, a British M. D., was published by the China Medical Missionary Association; the first edition in 1912 and the second edition in 1929. As a famous missionary doctor engaged in medical education in China in the early days, Cousland is an extremely important figure in the translation of Western medical books in modern Chinese history. He once served as the president of China Medical Missionary Association, the first national medical society in China, and was also a founder of the Compilation and Translation Department of China Medical Missionary Association. The medical books translated by him became authoritative in the early dissemination of Western medicine in China (39). Cousland wrote in the preface of *A Brief Introduction to Mental Illness* that “there is no medical book of insanity (madness) in China, and I would like to choose a book to render a simplified translated version for introduction. The book *Sanity in Everyday Practice* by Younger, designed for the diagnosis and treatment of mental illness by general practitioners, is concise but clear, comprehensive, coherent, and practical. I therefore chose it for my translation. As for those who specialize in the study of mental illness, this book is not enough.” In China, psychiatry developed later than other medical disciplines. Besides *A Brief Introduction to Mental Illness*, there were only four other monographs on psychiatry in the *Bibliography of the Republic of China 1911–1949: Natural Science: Medicine and Health* (40). The other four monographs are Huaying Ma’s *Lecture Notes on General Psychiatry* published in 1919 (41); Han’en Zhao’s *Psychiatry*, published by Commercial Press in Shanghai on 18 October 1929 (42); and Chi-liang Kwei’s *Modern Psychiatry* published in August 1932 in Shanghai and Beiping, with its copyright belonging to Crescent Moon Bookstore (43).

Chi-liang Kwei (1900–1956) was the first female psychiatrist in China, the first child psychiatrist, and one of the first generation of mental health experts who contributed to the development of modern psychiatry in China. She was funded by the Boxer Indemnity in 1922 to study at Wesleyan College in the United States. In 1925, she transferred to Hopkins University to specialize in psychiatry and obtained her M.D. in 1929. After returning to China in 1931, she successively worked with the Graduate School of Beijing Daoji Hospital (now the Sixth Hospital of Peking University), Hujiang University (now Shanghai University of Technology), and the Second Military Medical University, among others (44).

Hongqi Yao’s *Psychiatry* is actually an internal Lecture Notes for the fourth grade of the Medical College of Peiping University. There are four volumes of the Lecture Notes, namely, The Lecture Notes of the 20th Year (of the Republic of China) (1931) (45), the 21st Year (1932) (46), the 22nd Year (1933) (47), and the 23rd Year (1934) (48).

Han’en Zhao’s *Psychiatry* and Chi-liang Kwei’s *Modern Psychiatry*, written by the two local scholars, are the earliest professional works of psychiatry issued in modern China.

In terms of publication years, these four books are all later than *A Brief Introduction to Mental Illness*. Besides, Cousland, an authoritative figure who translated Western medical books in the early days, called the book “the first monograph on Psychiatry.” Therefore, it is quite credible that *A Brief Introduction to Mental Illness*, published in 1912, is the first monograph on psychiatry in China.

Depression was introduced as one of the six mental disorders (the other five are mania, paranoia, psychotic paralysis, dementia, and cretinism) and considered to be the most common and treatable of all mental illnesses, with a cure rate of about 70% in *A Brief Introduction to Mental Illness*. The book also states that any kind of depression would always have a gradual onset and degenerative, prodromal symptoms. It can be seen that this is quite different from the understanding of the pathology of depression today.

## Public Understanding of Depression in Early Days

In the previous section, we examined how depression was introduced and constructed in early modern China and found that the public cognition of depression was influenced and shaped by the mass media at that time, such as magazines, newspapers, and books, further leading to different forms of “rational oppression of irrational,” and formed the public stereotype about depression. The focus of this section is on the cultural images of depression in China in its early stages.

After the Meiji Restoration, Japan, on the one hand, “Westernized” and translated many depression-related texts from European and American psychiatric literature, and, on the other hand, integrated such knowledge into the psychological soil of East Asian society and culture. Obviously, localized knowledge borrowed from Japan was applied to China’s interpretation of the meaning of “depression” with a distinctive “polyphony” character. In this period in China, both among the nascent Chinese academic community of psychiatry and the general public, in medical texts, and literary and artistic works, depression was perceived and interpreted from both Western and Eastern perspectives. Thus, a new cognitive paradigm was created for the construction of localized psychiatry in China, where culture served both as a background and as a vehicle for the development of depression in neuropsychiatry. Unlike somatic diseases, mental disorders have relatively few specific objective indications; instead, they have a relatively rich narrative of wandering, experiential subjective sufferings. In early modern China, the pathology of depression was characterized by the psychological projection of suffering of the public, and the manifestation of the complexity of social context and uncertainties in life as a disease, and these characteristics also bring entanglement and confusion to the scientific construction of depression.

**FIGURE 2:**
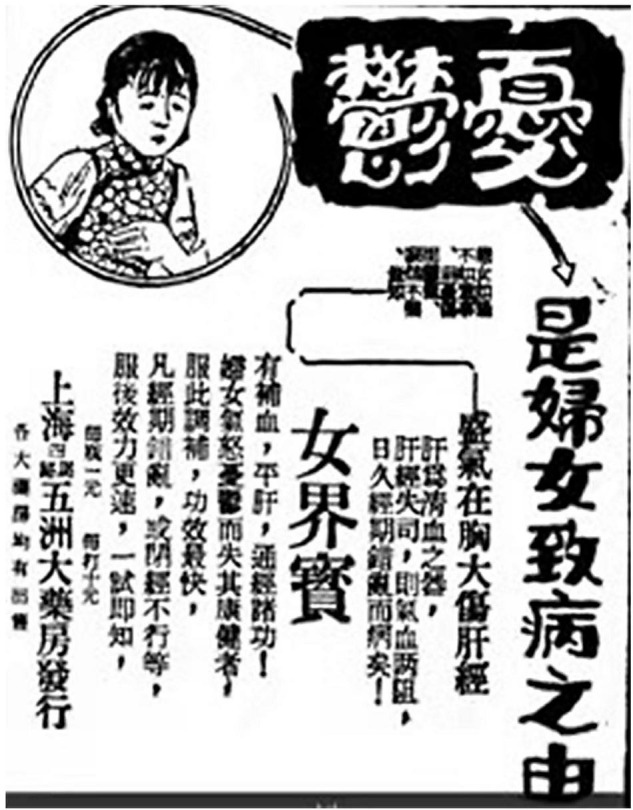
Advertisement of *Treasure of the Women’s World*. Source: *The News* (新闻报), 13 April 1930.

Modern Chinese people often associate melancholia with femininity, and depression was often regarded as a feminine disease. “Melancholia is the cause of women’s illness. If a woman is unhappy, she will be depressed. It must be pointed out that her excessive *Qi* in the chest hurts her liver meridian. The liver is the instrument for clearing blood, and if the liver meridian is out of control, then both *Qi* and blood will be blocked. If the condition persists, the menstrual period would be disrupted, and she would fall sick!” reads the advertisement sponsored by the Five Continent Dispensary in the healthcare column of the *News* published on 13 April 1930 (49), and in the advertisement entitled *Treasure of the Women’s World*, published consecutively in the *Times* on 20–24 April 1930 (50–54) (Figure 2). It was pointed out that women’s anger and depression are the causes of gynecological diseases. This case shows that people had already understood the relationship between emotional state and physical diseases at that time, and knew that psychological problems could lead to physiological problems, which could be regarded as the predecessor of the concept of psychosomatic diseases.

The depression in modern China is often regarded as a symbol of the intellectual youth, who were worried about the country and the people, and it is the label of this special group of youth. Under the background of national crisis and internal and external troubles, an individual’s depression is linked to the fate of the country. With social unrest and turmoil, young people encountered challenges in their education and employment, and their melancholia became a serious social problem, which raised concerns among scholars, who wrote articles to express their worries. In *Unwanted Melancholia: One of the Treatments for Youth’ Disease*, the author wrote:

I think most Chinese youths born in this era are afflicted by melancholia. Although they shout the slogan of progress, claim that they had god-gifted energy, and are forced by reality to move forward, they seem to always be bedridden of self-doubt, and could never struggle out of the types of sentimental young lords and ladies. Some are severely ill, walking slowly and sitting with unrest (55).

Due to depression, many young people look at everything in the world with an unhappy face as if everything were in shade or they were crushed by a rock on their chest. Sadness, anger, and other unnecessary emotions dominate their lives. They are wary of this world, although do not hate it, and live their lives as if they were in another realm. Some scholars got upset and criticized these young people’s depression as a disease-free moan and pointed out that they might spread depression to their peers by the influence of their demeanor. They believed that young people at that time would naturally become depressed because they lived in a restless era, encountered setbacks in their personal growth, had witnessed many cases of others’ failure, and heard the melancholy groans of their predecessors. They warned that when the depression of one person or some people was assimilated into many people, an “epidemic of the times” would break out, not only destroy the bright future of young people who make unnecessary sacrifices for nothing, but also, more importantly, undermines the future of the nation.

Clearly depression in young people was associated with worry about the strength of the nation and the endurance of the nation. Scholars warned that young people’s depression had threatened the development of the national economy and hindered the process of social evolution and that, in light of the consequences for the nation and society, attention must be paid to their malaise and to stop the deterioration of the situation. The depression of young people was even linked with the survival of the nation, and it was believed that the depression of young people is not only their great misfortune but also that of the nation.

It was emphasized that young people should take responsibility for their depression. As for the way to overcome depression, the following proposals were made, namely, keep fit in body and mind; be discernible but do not get obsessed with things; be optimistic; work hard without caring for reward; no worry about failure, maintain curiosity, and be forward-looking; and make use of leisure life to avoid loneliness and the subsequent sadness.

In his article *On the Melancholia of Young People*, Ping Jiang argues that the reason young people got confused and suffered from depression was that the complex and treacherous social environment in China at that time made it difficult for young people who were not deeply involved in the world yet, to make choice. This shows his sympathy for the young people. He wrote that “the opinions in China have not yet been consistent. Some people advocate prioritizing efforts on survival and competing with the enemy; some people believe in mutual assistance, tolerance, and accommodation with external forces. Take the way to save the nation as an example. There are a range of propositions, advocating saving it by conscience, learning and study, science and technology, entertainment, or reading and chanting Buddhist sutra. It is confusing even for sophisticated, older people to see the true face from behind the masks, let alone a young man/woman!” (56).

The author gives his prescription to fight depression. He calls the young to believe that human beings are evolving and the youth should understand their responsibilities in evolution so that they will not regard their lives as worthless. The author believes that at the same time, young people, although should be ambitious and think about how to save the nation, should talk, laugh, jump, and sing in their daily life, instead of reading books all day long.

Hypochondria was often translated as *忧郁症 Youyuzheng* in early literary works. The word hypochondria, as is known to well-educated people, reflects preoccupation with imagined problems with health. For example, *The Hypochondria*, a collection of dozens of short essays published in the *London Journal* between 1777 and 1783, written by James Boswell, was translated into *The Melancholy Patient* when it was introduced to China (57). Dr. Beili, professor of literature at Stanford University, has verified the authorship of this collection. In addition, Dafu Yu, a famous author at that time, also wrote in the preface of his novel *Sinking* that “this work describes the psychology or hypochondria of sick youth, revealing the depression of modern people, caused by the (unsatisfied) sexual desire and conflict between body and soul” (58). The characters described in the novel are often literati with lung disease and depression, suffering from self-blame, sorrow, melancholy, nothingness, and imagination of sexual intercourse caused by unsatisfied sexual desire. In *Paratypical Melancholia* in *Novel: Shanghai 1934* (59) published in 1935, a tragic character, *Fengzi*, is such an image. *Fengzi* is depicted as a pale-face, thin-cheeked, and dry-eye intellectual suffering from depression and tuberculosis.

The depression was also regarded as a popular symbol of modern urban life. Depression in literary and artistic works is often accompanied by mental overwork, entangled romantic relationships, debauchery, lust, weakness, loneliness, pain and sufferings of separated lovers, addiction to the pleasures of music, women, hunting, and racing. For example, melancholia was taken as the theme in the article *One of the Sketches of Modern Urban Life: Epidemic Melancholia* published on 23 November 1934, in *Min Pao Daily 民报* (60), the novel *Women, Coffee, and Melancholia* published in *the Spring* magazine in 1935 (61), and the poem *Melancholia* published in the *Shilin Bimonthly* in 1936 (62). Obviously, in the Republic of China period, people had already known about this disease. In poems, novels, and essays, melancholia is often accompanied by love, coffee, jazz, beer, and wine, the elements of modern life at that time, alongside the representation of resentment, boredom, restraint, sadness, laziness, laziness, and loneliness. Melancholia has become a special form of sadness that possesses superiority unique to the rich and idle upper class. Through the shaping of these literary and artistic works, in the public impression at that time, depression was not so much a disease, but a morbid and distorted “gray” state of life common in “literary youth” and the upper class.

The expression of disease does not exist in a vacuum irrelevant to the social environment. So are mental diseases. In the early period of the introduction of depression into Chinese society, the pattern of depression presentation also had the characteristics of the times. In 1934, an article published on page 23 of *Shen Bao 申报* posits that mental disorders such as suicide, melancholia, women’s hysteria, neurasthenia, and madness were prevalent in society at that time and that in the modern, civilized world, almost everyone had the possibility of suffering from mental illness. In the then-Chinese society, under panic induced by economic depression and prevalent employment difficulties, ill-health of mind had become common. Melancholia, suicide, madness, and other behaviors are the ways used by the unemployed to express their protests against the tragic social context and their pathetic failure (63). We have found that at that time people around 30 years old have a high risk of melancholia. If one wants to recover from melancholia, he/she needs to absorb more nutrients and reduce labor, especially mental work. However, for those who did not have enough food and clothing despite daily work that exceeds more than 10 h, it was simply impossible.

## Notes

Advertisements for antidepressants and descriptions of depression began to appear in Chinese and English newspapers and in journals in the late period of the Qing Dynasty (1616–1911) and the Republic of China (1911–1949), as well as in early psychiatric books. Such advertisements and descriptions reached their peak in the 1930s, in the time frame of early modern China. The Western concept of depression was quietly implanted in the country through the abovementioned media and, to a certain extent, shaped the public cognition of depression in China. This study adopts a text analysis method, which uses first-hand historical documents as a carrier, and finds the results as follows.

## Data Availability Statement

The original contributions presented in the study are included in the article/supplementary material, further inquiries can be directed to the corresponding author.

## Author Contributions

JB: conception and design of study, conductor of the study, acquisition of materials, analysis and interpretation of materials, and drafting the manuscript. JL: help translate. DZ: approval of the version of the manuscript to be published. All authors contributed to the article and approved the submitted version.

## Conflict of Interest

The authors declare that the research was conducted in the absence of any commercial or financial relationships that could be construed as a potential conflict of interest. The reviewers YT, YW, and YFW declared a shared affiliation with the authors to the handling editor at the time of review.

## Publisher’s Note

All claims expressed in this article are solely those of the authors and do not necessarily represent those of their affiliated organizations, or those of the publisher, the editors and the reviewers. Any product that may be evaluated in this article, or claim that may be made by its manufacturer, is not guaranteed or endorsed by the publisher.
